# Discovery of a *cis*-regulatory element *SaeM* involved in dynamic regulation of synergid-specific *MYB98*

**DOI:** 10.3389/fpls.2023.1177058

**Published:** 2023-05-08

**Authors:** Prakash Babu Adhikari, Shaowei Zhu, Xiaoyan Liu, Chen Huang, Liyang Xie, Xiaoyan Wu, Jiale He, Nobutaka Mitsuda, Benjamin Peters, Lynette Brownfield, Shingo Nagawa, Ryushiro Dora Kasahara

**Affiliations:** ^1^ Key Laboratory of Horticultural Plant Biology (Ministry of Education), Huazhong Agricultural University, Wuhan, China; ^2^ College of Life Science, Fujian Agriculture and Forestry University, Fuzhou, Fujian, China; ^3^ Horticultural Plant Biology and Metabolomics Center (HBMC), Fujian Agriculture and Forestry University, Fuzhou, Fujian, China; ^4^ Bioproduction Research Institute, National Institute of Advanced Industrial Science and Technology (AIST), Tsukuba, Japan; ^5^ Department of Biochemistry, School of Biomedical Sciences, University of Otago, Dunedin, New Zealand; ^6^ International Research Organization for Advanced Science and Technology (IROAST), Kumamoto University, Kumamoto, Japan

**Keywords:** *MYB98* promoter, *SaeM*, cis-element, synergid cell, dynamic regulation

## Abstract

*MYB98* is a key regulator of the genetic network behind pollen tube attraction toward the female gametophyte. *MYB98* is specifically expressed in the synergid cells (SCs), a female gametophyte component cells specialized for pollen tube attraction. However, it had not been clear how exactly *MYB98* achieves this specific expression pattern. In the current study, we have determined that a normal SC-specific expression of *MYB98* is dependent on a 16-bp-long *cis*-regulatory element, CATTTACACATTAAAA, freshly named as the “***S*** *ynergid-specific* ***A*** *ctivation* ***E*** *lement of* ***M*** *YB98*” (*SaeM*). An 84 bp fragment harboring *SaeM* in the middle was sufficient to drive exclusively SC-specific expression. The element was present in a significantly large proportion of SC-specific gene promoters and in the promoter of *MYB98* homologous genes in the Brassicaceae (*pMYB98*s). Significance of such family-wide *SaeM-like* element conservation in exclusive SC-specific expression was confirmed by the Arabidopsis-like activation feature of *Brassica oleracea–*derived *pMYB98* and absence of such feature of *pMYB98* derived from a non-Brassicaceae member *Prunus persica*. Additionally, the yeast-one-hybrid assay showed that the *SaeM* can be recognized by ANTHOCYANINLESS2 (ANL2) and DAP-seq data further suggested for additional three ANL2 homologs targeting the similar *cis-*element. Overall, our study has concluded that *SaeM* plays a crucial role in driving exclusively SC-specific expression of *MYB98* and strongly suggests for the involvement of ANL2 and its homologs in its dynamic regulation *in planta*. Future study on the transcription factors is expected to shed more light on the mechanism behind the process.

## Introduction

The female gametophyte (FG) is an important tissue of sexual reproduction in plants. The FG develops from a single meiotic product with several rounds of mitotic nuclei division being followed by cytokinesis. A typical mature FG comprises seven cells: two synergid cells (SCs; haploid) at the micropylar end, three antipodal cells (ACs; haploid) at the chalazal end, a central cell (CC; homodiploid) at the middle and an egg cell (EC; haploid) sandwiched between the SCs and CC. During fertilization, the EC and CC take the central stage as they develop into embryo and endosperm, respectively, after their independent fertilization with two sperm cells released from the pollen tube (PT) (Drews and Koltunow, 2011; Adhikari et al., 2020). However, it is the SCs that are specialized and quintessentially involved in attracting the PT to the FG followed by its reception. (Higashiyama, 2002; Kessler and Grossniklaus, 2011). The PT follows the traces of the molecular cues secreted by SCs, which mainly include cysteine rich proteins (CRPs) (Okuda et al., 2009; Takeuchi, 2021). Secretion of such cues is largely regulated by an SC-specific master regulator transcription factor (TF), *MYB98* (Kasahara et al., 2005; Punwani et al., 2007; Kasahara, 2018). *MYB98* expression is initiated during the cellularization process and is later restricted exclusively to the SCs in the mature FGs (Kasahara et al., 2005; Zhang et al., 2020; Susaki et al., 2021). MYB98 is involved in the development of filiform apparatus and the regulation of crucial genes directly involved in PT attraction (Kasahara et al., 2005; Punwani and Drews, 2008). There have been some extensive studies on the genetic networks involved in PT guidance downstream of MYB98 (Punwani et al., 2007; Punwani et al., 2008). A recent study reported that post-transcriptional pre-mRNA splicing of *MYB98* and CRP is seriously hindered upon mutation of the spliceosome subunit encoding genes *PRP8A* and *PRP8B*, thereby negatively affecting the synergid fate and ovular PT-attraction (Kulichová et al., 2020). However, even after nearly two decades of the discovery of its involvement in PT attraction (Kasahara et al., 2005), the molecular factors behind *MYB98* regulation remain largely unknown. The central-cell specific TFs CCG and CBP1 positively affect *MYB98* expression in SCs, but the process behind this is not fully clear (Li et al., 2015). *MYB98* expression in the CC is actively repressed by the EAR motif harboring MADS box TF AGL80 binding to the CArG boxes in the *MYB98* promoter (*pMYB98*). Ectopic expression of the *AGL80* in SCs leads to the repression of native *MYB98* and the ovules exhibit *myb98* mutant-like PT-guidance defect (Zhang et al., 2020).

Here, we report the discovery of the **S** ynergid-specific **A** ctivation **E** lement of ***M*** *YB98* (*SaeM*) in the Arabidopsis-derived *MYB98 promoter* (*pMYB98*), which is crucial for its exclusively SC-specific expression. The element and associated regulatory features are conserved among *pMYB98*s of Brassicaceae members. Furthermore, a homeodomain member ANTHOCYANINLESS2 (ANL2) binds to the *cis-*fragment harboring the *SaeM* potentially mediating its dynamic regulation.

## Results

### Identification of pMYB98 region crucial for driving synergid-specific expression

**Figure 1:**
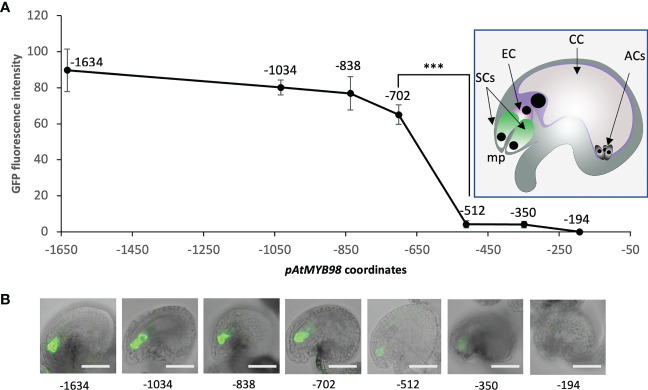
A 191-bp-long *pMYB98 cis*-fragment deletion significantly reduces the reporter signal. **(A)** SC-specific fluorescence intensity quantified in the ovules of plants transformed with the reporter constructs driven by varying lengths of *pAtMYB98* (***p ≤ 0.001, DMRT; *n* = 20). *Inset:* Typical Arabidopsis ovule with its component cells denoted. **(B)** Representative CLSM images of the ovules derived from the bashed *pMYB98::GFP* transgenic lines. Values represent mean ± SE; *n* = 15; scale bar = 50 μm; SCs, synergic cells; EC, egg cell; CC, central cell; ACs, antipodal cells; mp, 'micropyle.

As a first assessment to identify the regions of *pMYB98* that contribute to the SC expression of *MYB98*, a series of promoter deletions from both the 5′ and 3′ ends were screened for activity based on reporter (GFP) expression in Arabidopsis ovules. In both cases, the numbering of the bases was relative to the translation start site (ATG). The *pMYB98* 5′-deletion reporter series showed that the reporter signal drops upon deletion of a −838 to −702 region, a −702 to −512 region, and a −350 to −194 region with only the middle one displaying statistically significant fluorescence signal changes. Furthermore, unlike some of the reporter lines harboring −350 bp *pMYB98* fragment, none of the reporter lines with −194 bp fragment exhibited fluorescence signal (**Figure 1**). Consistent with these results, a 3′-deletion series showed that deletion of either of two specific promoter regions, −615 to −487 bp and −251 to −121 bp, brought drastic drop in the reporter signal (**Supplementary Figure S1**). Combined, these observations strongly indicated that a 191 bp region between −702 and −512, the deletion of which exhibited a steep drop in the reporter signal, harbors one or more sequence elements that are crucially important for SC-specific expression of *MYB98.* Furthermore, a 156 bp region between −350 and −194 bp can drive weak yet quantifiable SC-specific expression.

### Brassicaceae members harbor uniquely conserved sequence at the 3′-end of the functionally active promoter region

**Figure 2:**
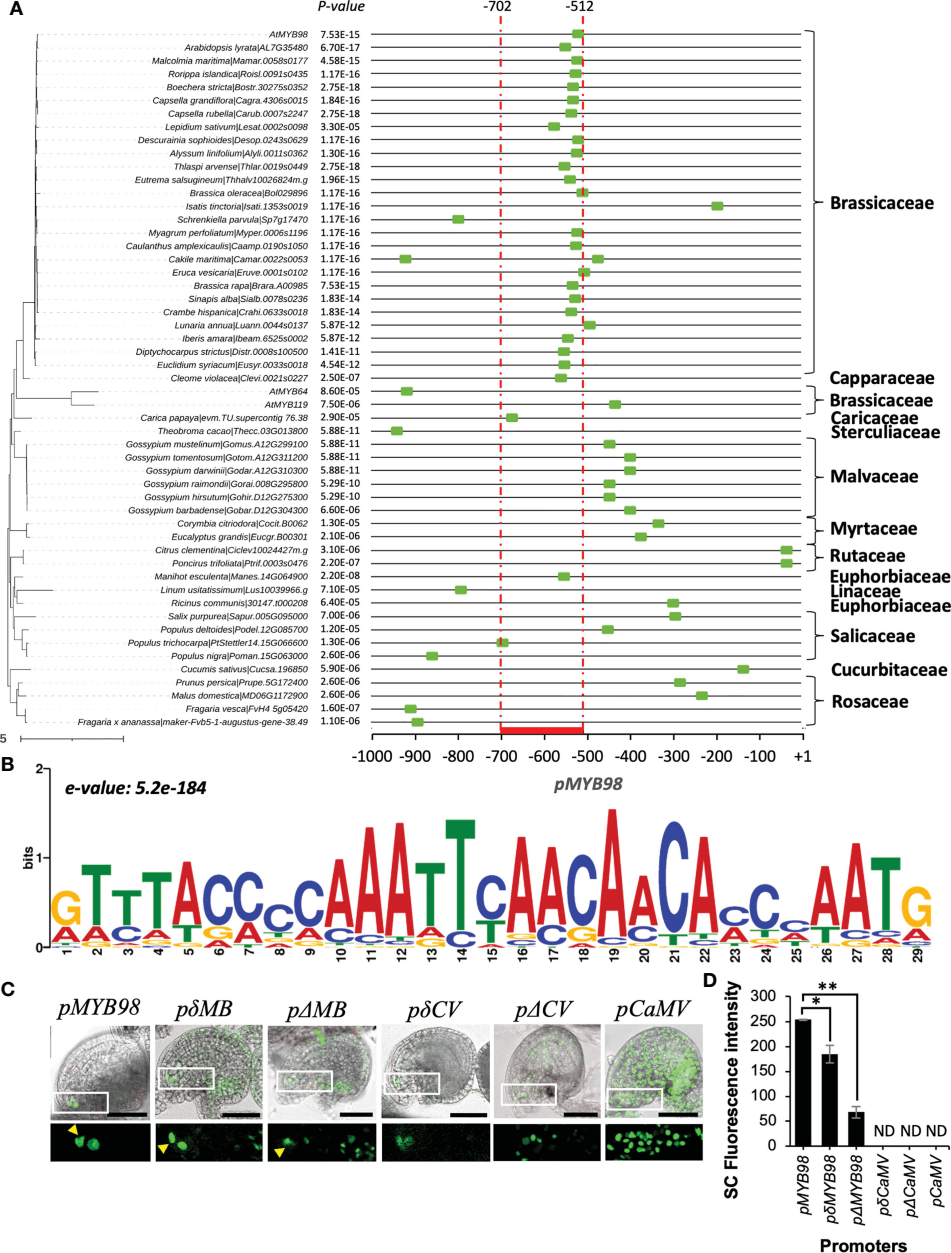
Assessment of motif conservation among *MYB98* homologs and putative TF binding sites within the functional active 191 bp region (in reference to *A. thaliana*). **(A)** The most conserved motif within the 1 kb promoter region of *pAtMYB98* region (marked with a green box at the x-axis). The phylogenetic tree on the left is the subtree of phytozome derived 108 *pMYB98* homologs and *AtMYB* sub-group 25 members (check **Supplementary Figure S2** for reference). **(B)** Position-specific scoring matrix of the conserved motif. (e-value: 3.9e-183) in *pMYB98* from 51 Species. **(C)** Expression of *H2B-GFP* driven by the intact (*pMYB98*), conserved motif deleted (*pδMB*), and whole functional motif deleted (*pΔMB*) *MYB98* promoters along with the conserved motif added (*pδCV*) and whole functional region added (*pΔCV*) *CaMV* promoters. The arrowheads point SCs. **(D)** GFP intensity at SCs of the aforementioned *pMYB98* fragment deletion and addition lines. (ND = not clearly distinguishable; *p ≤ 0.01, **p ≤ 0.001, Student’s t-test; *n* = 9). Scale bar = 50 μm.

*MYB98* is activated during FG development to drive SC cell fate with its expression precisely restricted to the SCs in mature FGs (Kasahara et al., 2005; Susaki et al., 2021). Based on this, we hypothesized that there could be a conserved motif in *pMYB98* homologs responsible for the specific expression pattern. To test this, we retrieved 1 kb sequence of respective 5′-upstream region of the putative *MYB98* homologs from the Phytozome database (Goodstein et al., 2012) and selected only those closely clustered with the AtMYB subgroup 25 members (*AtMYB98*, *AtMYB64*, and *AtMYB119*) for the phylogenetic footprinting (**Figure 2A** and **Supplementary Figure S2**) (see material and methods section for details). A native motif search among the promoters of the 51 *MYB98* homologs along with *AtMYB64* and *AtMYB119 via* MEME (Bailey et al., 2009) revealed that the most conserved 29 bp long motif falls at the 3′-end of the functionally active Arabidopsis *pMYB98* with an 8 bp extension at the 3′ end (*e*-value: 5.2e-184). The members of Brassicaceae have a distinctive pattern for both the site and degree of motif conservation in comparison with *pMYB98* from other plant families. Except for two, all Brassicaceae derived promoters harbor the motif within −700 to −400 bp (**Figures 2A, B**), suggesting that the transcriptional regulation of *MYB98* could have been conserved among Brassicaceae members with some variations. While there is more variation in other plant families, the motif was still commonly found within the 1,000 bp region analyzed with the *MYB98* homologs from the Malvaceae (Wu et al., 2022), a close evolutionary relative of the Brassicaceae being the most similar, although the motif site was located at further downstream (**Figure 2A**).

### Loss of conserved motif leads to the loss of exclusive synergid-specificity of pMYB98

To ascertain whether the putatively conserved region is crucial for driving SC-specific expression of *pMYB98*, we carried out a deletion/addition reporter expression assay. Unlike the intact 1.5k promoter (*pMYB98*) lines, which normally exhibit SC-specific expression in the mature FGs, the reporter lines lacking the conserved motif (*pδMYB98*: −559 to −509 bp deleted) lost this specificity, along with a slight but significant drop in GFP intensity. However, when the entire 191 bp functionally active region was deleted (*pΔMYB98*; −702 to −509 bp deleted), the GFP signal dropped steeply along with the loss of SC-specific activation (**Figures 2C, D**). We additionally checked whether either the conserved region or the whole 191 bp fragment drives SC-specific expression when incorporated in the middle of the 346 bp constitutive *pCaMV35S* promoter at 241 bp upstream of the ATG (*pδCaMV* and *pΔCaMV*, respectively). On its own the *pCaMV* region leads to GFP expression throughout the ovule, making it difficult to distinguish the SC (**Figure 2C**). The addition of either the conserved region or the 191 bp region to *pCaMV* did not provide SC-specific expression, although there was reduced expression within the ovule, especially for *pδCaMV*. Additionally, the fluorescence intensities provided by both *pδCaMV* and *pΔCaMV* were much lower compared with that of the intact *pMYB98* driven GFP (**Figures 2C, D**). Overall, these observations on the *cis*-element addition/deletion reporter lines strongly indicated that the conserved motif is likely involved in overall repression and the 191 bp region of *pMYB98* likely harbors additional important *cis-*element crucial for SC activation. To further define the crucial *cis*-elements within the functionally active *pMYB98* region, we carried out reporter assays with sub-fragment combinations and mutations of putative *cis*-elements.

### An 84 bp fragment within pMYB98 is sufficient for its SC-specific activation

**Figure 3:**
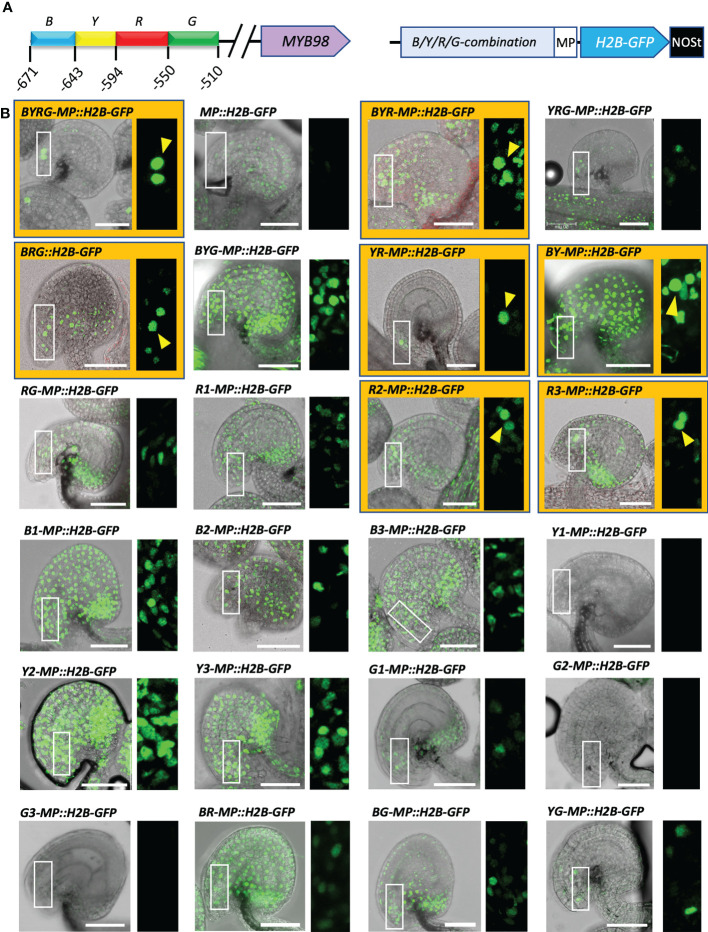
A short 84 bp fragment located at the middle of the functionally active *pMYB98* region is sufficient for the exclusive SC-specific expression. **(A)** *pMYB98 cis* fragments (B, Y, R, and G) used in the study and their locations upstream of ATG (+1) (*left*) along with the diagram representing a typical construct used in the study (*right*). **(B)** Representative CLSM images of ovules derived from the reporter lines of the B, Y, R, and G fragments along with negative control (MP). Images of the ovules with distinguishable SCs are highlighted with orange color and distinguishable SCs are pointed with arrowheads. Check **Supplementary Movie S1** for the associated z-stack-derived movies of ovules with distinguishable SCs. Scale bar = 50 μm; MP, minimal promoter.

Based on the observations made on the 5′- and 3′-deletion series (**Figures 1**, **S1**), we selected a 169 bp region (−678 to −510) of *pMYB98* and divided it into four sub-fragments, annotated as Blue (B, 45 bp), Yellow (Y, 40 bp), Red (R, 44 bp), and Green (G, 40 bp) (in order from 5′ to 3′) (**Figure 3A**). The fragments were assembled in all possible 2-4 fragment combinations along with the 1-3 repeated combinations of each sub-fragments (24 combinations in total including negative control construct). The prepared combinations were cloned upstream of *CaMV* minimal promoter (MP) independently to drive H2B-GFP expression (**Figure 3A**). The complete fragment (*BYRG-MP::H2B-GFP*) provided strong SC-specific GFP fluorescence, while the minimal promoter on its own (*MP::H2B-GFP*) provided weak expression in sporophytic cells but not in the SCs (**Figure 3B**). We then considered the role of each of the fragments.

The B-fragment appears to have a non-cell–specific role in transcriptional activation as the removal of the B-fragment in *YRG-MP::H2B-GFP* resulted in a decrease in expression and containing only the B-fragment (*B1-MP::H2B-GFP*, *B2-MP::H2B-GFP*, and *B3-MP::H2B-GFP*) along with some other fragments containing B-fragment (e.g., *BYG-MP::H2B-GFP* and *BY-MP::H2B-GFP*) had strong fluorescence throughout the ovule with too prevalent signals in the sporophytic cells (**Figure 3B**).

The Y-fragment and the R-fragment appear to work in combination to both provide some transcriptional activation in SCs and also play a key role in restricting expression to the SC. When the Y-fragment was removed (*BRG-MP::H2B-GFP*), SC-expression was retained but had reduced intensity and GFP was no longer SC-specific. When the R-fragment was absent (*BYG-MP::H2B-GFP*), SC fluorescence could not be distinguished (**Figure 3B**). Additionally, repeats of the R- fragment (*R2-MP::H2B-GFP* and *R3-MP::H2B-GFP*) lead to GFP fluorescence in multiple FG cell types in some cases (**Supplementary Figure S3**). Notably, a combination of just the Y- and R- fragments (*YR-MP::H2B-GFP*) resulted in SC-specific fluorescence, indicating that the region covered by R and Y contains cis-elements that restrict expression to the SCs within the ovule (**Figure 3B**).

When the G-fragment is removed (*BYR-MP::H2B-GFP*), SC fluorescence is retained, but the GFP signal is no longer restricted to the SCs. However, the G-fragment on its own (*G1-MP::H2B-GFP*, *G2-MP::H2B-GFP*, and *G3-MP::H2B-GFP*) did not increase GFP levels over *MP::H2B-GFP*. This suggests that the G-fragment contains cis-elements that help provide SC-specificity but do not assist in transcriptional activation (**Figure 3B**).

Overall, assessment of the fragment combination driven expressions strongly indicated that the 94 bp long YR-fragment is key to driving exclusively SC-specific expression. The SC expression is augmented by upstream cis-elements located in the B-fragment and further restricted by downstream cis-elements located in the G-fragment (**Supplementary Table S1**). To further narrow down the TF-binding sites crucial for SC-specific *pMYB98* activation, we carried out a repetitive *cis*-element mutation assessment.

### Discovery of the 16-bp-long synergid-specific activation element of MYB98

**Figure 4:**
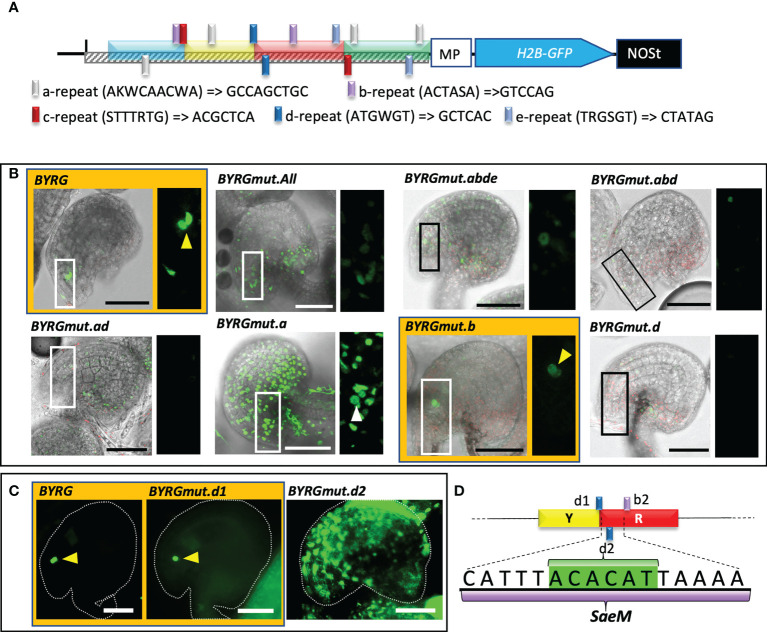
Mutation of two 6 bp repeats within the BYRG-fragment effectively silences the reporter expression. **(A)** A schematic diagram of a reporter construct harboring functionally active *pMYB98* with the relative sites of the repeat elements targeted for mutation. Native and the substituted bases are shown with the repeat element legends. **(B)** Representative CLSM images of the ovules derived from the reporter lines of the negative control (MP) as well as the normal and mutated BYRG-fragments. **(C)** Epifluorescence images of positive control and independent d-repeat mutant-derived ovules. **(D)** Determination of *Synergid-specific activation element of MYB98* (*SaeM*) harboring a d-repeat (highlighted in green) between upstream localized d-repeat (*d1*) and downstream localized b-repeat *(b2*). Images of the ovules with distinguishable SCs are highlighted with orange color and distinguishable SCs are pointed with arrowheads. *Scale bar = 50 μm MP, minimal promoter*.

Since the intact 191 bp functionally active fragment was necessary and sufficient to drive SC-specific expression and the fragments had differing roles, we assumed that there could be multiple-binding sites (*cis*-elements) for key TF involved in the transcriptional regulation of *MYB98.* A manual assessment for the putatively repeated short sequences within the full functional element revealed five repetitive elements, which were annotated as “a” (AKWCAACWA, four copies), “b” (ACTASA, two copies), “c” (STTTRTG, two copies), “d” (ATGWGT, two copies), and “e” (TRGSGT, two copies) (**Figure 4A**). To test the function of the repeats, each repeat was mutated to GCCAGCTGC, GTCCAG, ACGCTCA, GCTCAC, and CTATAG, respectively, to assess its effect on SC-specific activation (**Figure 4A**). Among the different combinations of reporters with repetitive element mutations, only the intact and b-repeats mutated fragments brought exclusively SC-specific expression, even though mutation of the b-repeats led to the reduction in GFP signal (**Figure 4B**). Interestingly, all combinations that included d-repeats mutation, including mutation of the d-repeats alone, exhibited either no expression at all or no expression at SC (**Figure 4B**), suggesting that a transcriptional activator binds to the d repeat. Two copies of the 6 bp d-repeats are closely located (−611 to −595), one of which is at the Y–R junction, while the other is within the R fragment (**Figure 4A**). The observation partly comes in agreement with the fragment combination observation we made earlier, which showed that unlike B-fragment alone, the fragment lacking YR exhibited a much-reduced fluorescence signal (**Figure 3B**).

Interestingly, the mutation of the d-repeat at the Y–R junction alone has little to no effect on the SC-specific activation of the fragment, while mutation of the d-repeat located downstream in R-fragment led to ovule-wide GFP expression with no distinguishable signal at SCs (**Figure 4C**), which was very similar to the observation we made earlier with the BYG combination (**Figure 3B**). Since four concatemers of d-repeats alone could not drive the GFP expression (data not shown), we presumed that it is not sufficient to drive SC-specific expression, and instead, its mutation most likely has disrupted the putative binding site of a key TF responsible for such phenomenon. However, the mutation in b-repeats, one of which falls downstream of the d-repeat within the R-fragment (−589 to −584), could still exhibit SC-specific expression (**Figures 4A, B**), suggesting that the 16-bp-long *cis*-region sandwiched between the d-repeat at Y–R junction and b-repeat at R-fragment (−577 to −592), catttACACATtaaaa, is essential for overall as well as SC-specific activation of the 169-bp fragment of *pMYB98* (**Figure 4D**). We annotated this sequence as a “***S*** *ynergid-specific* ***A*** *ctivation* ***E*** *lement of* ***M*** *YB98*” (*SaeM*).

### Synergid-specific activation element of MYB98–like element is enriched in SC-specific gene promoters and highly conserved among Brassicaceae-derived pMYB98s

**Figure 5:**
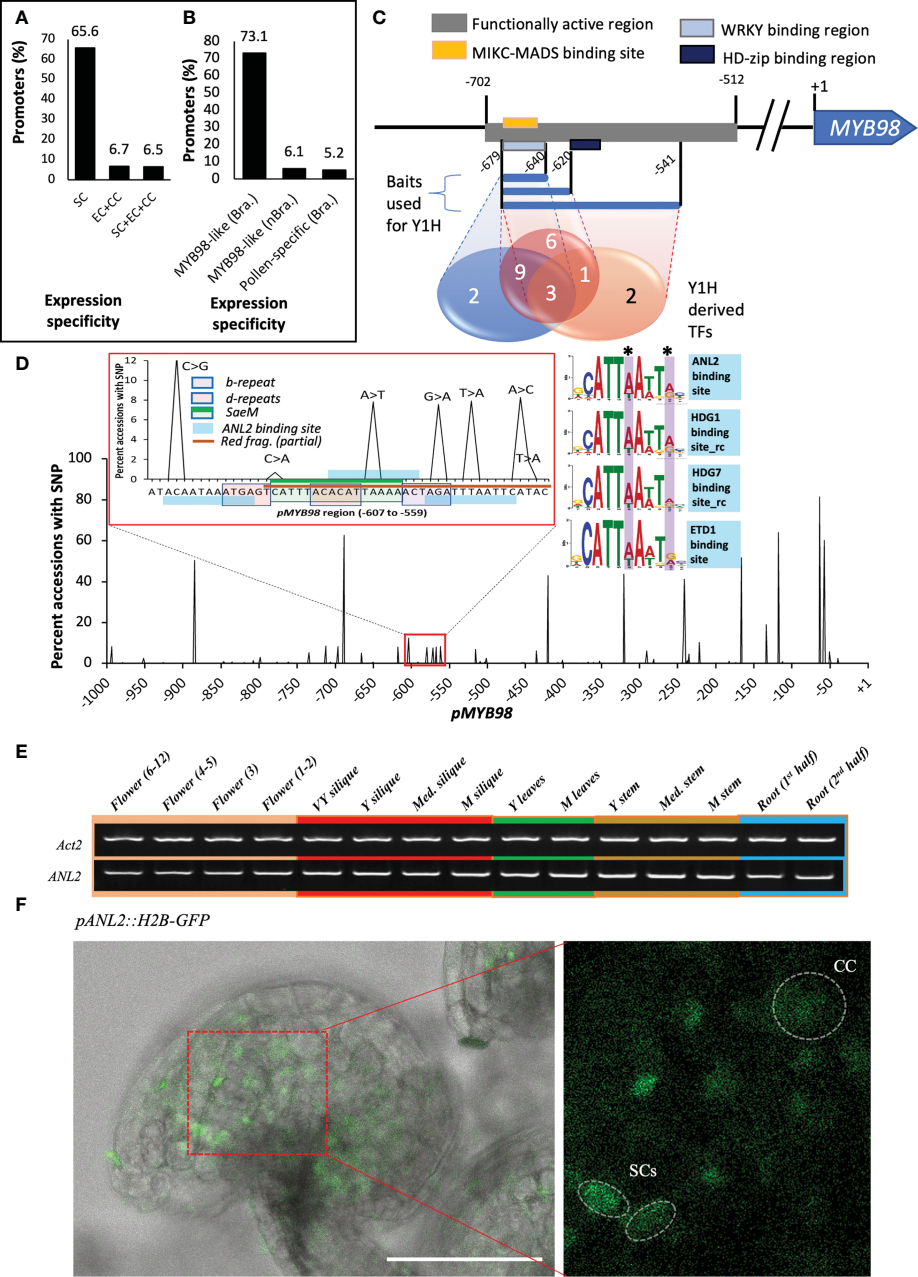
*SaeM* is conserved among Brassicaceae members and is recognized by ANL2 and its homologs. **(A)** An excessively higher proportion of SC-specific gene promoters harbor *SaeM*-like element as compared with those expressed either at all female gametophyte component cells or exclusively at CC and EC. **(B)** A significantly high proportion of Brassicaceae-derived *pMYB98* harbor *SaeM-like* element as compared with their non-Brassicaceae counterparts and Arabidopsis derived pollen-specific gene promoters (-ve control). **(C)** TF-binding sites predicted by the PlangRegMap (in square boxes) and the number of TFs derived from yeast-1-hybridization (Y1H) with three independent overlapping fragments as baits (in Venn diagram). **(D)** SNP frequency within 1 kb of *pMYB98* among 1,135 Arabidopsis accessions. The *SaeM*, within/nearby repeat-sites used for mutation studies, and ANL2-binding sites are highlighted in the magnified graph in inset. The web logos of respective recognition sites of ANL2 and its homologs (HDG1, HDG7, and ETD1) are shown on the side marking and highlighting the base positions with SNPs. **(E)** *ANL2* is expressed in all plant tissues assessed in Arabidopsis. **(F)** Weak GFP-signals at SCs and CCs in *pANL2::H2B-GFP* mature ovule (retrieved from Flower stage 3). GFP intensity was enhanced for visualization purpose; check the associated z-stack-derived movie in **Supplementary Movie S3**. Scale bar = 50 μm. SC, synergid cell; CC, central cell; EC, egg cell.

To ascertain whether *SaeM* is conserved among synergid-specific promoters, we compared promoter regions between SC and non-SC gene pools. We defined the *SaeM*-aligned respective sequence region as the *SaeM*-like element for each promoter sequence. Among 64 exclusively SC-expressed genes, about 66% harbored a *SaeM*-like element within their 1 kb promoter region. Among them were those that had been experimentally verified to be SC-specific, such as *MYB98* (Kasahara et al., 2005; Punwani et al., 2007) *LURE*s (1.1, 1.2, 1.3, 1.4, 1.7, and 1.8) (Takeuchi and Higashiyama, 2012; Zhong et al., 2019), *LORELEI* (Noble et al., 2022), *DD11* and *DD18* (Punwani et al., 2007). (**Figure 5A** and **Supplementary Table S2**). However, the *SaeM*-like element was found in the promoters of only about 7% of the genes expressed in either all FG component cells (> 0 abundance at all SC, CC, and EC replications; 138 genes) or exclusively at EC and CC (null expression value at all SC replication but > 0 abundance at all EC and CC replications; 163 genes) (**Figure 5A** and **Supplementary Table S2**-**S4**). Such occurrence strongly indicated that *SaeM* element is most likely to be involved in the activation of the associated genes in the SCs and their simultaneous non-activation in non-SCs.

Our phylogenetic footprinting assessment showed uniquely conserved motif pattern for the Brassicaceae members derived *pMYB98*s as compared with non-Brassicaceae derived counterparts (**Figure 2A**). In agreement to such assessment, we found that *SaeM-like* element is present in the significantly higher numbers of the Brassicaceae-derived *pMYB98*s (73% of 26 genes). On the other hand, only non-significant proportion of non-Bassicaceae derived *pMYB98*s (6% of 82 genes) appear to harbor the element which was very similar to that of the pollen-specific gene promoters retrieved from the study of Honys and Twell (2003) (5% of 381 genes) (**Figure 5B**; **Supplementary Table S5** and **S6**). It is also noteworthy that *AtMYB64*, which is reportedly expressed at SCs along with the relatively weaker expression at other FG component cells (Rabiger and Drews, 2013), also harbor a *SaeM-like* element in its promoter.

To determine potential TFs binding to the *SaeM* and the wider region, we carried out a Yeast-one-hybrid assay (Y1H) assessment.

### ANL2 and homologs show binding potential to the Synergid-specific activation element of MYB98

We took the 5′-deletion series derived functionally active 191 bp *pMYB98* fragment for the prediction of potential TF-binding sites and associated TFs using PlantRegMap (Tian et al., 2019) (threshold *p*-value ≤ 1e−4). The −671 to −595 region was predicted as the potential binding pocket for WRKY, HD, and MIKC-MADS TFs (**Figure 5C** and **Supplementary Table S7**). Based on the predictions, three independent bait constructs were prepared using the 40, 60, and 139 bp fragments of overlapping sequences for the Y1H assay. The screening was performed against an Arabidopsis TF prey library (Mitsuda et al., 2010). Among the three, 60 bp fragments gave the highest number of candidate TFs (19 in total) followed by 40 and 139 bp fragments (14 and 6, respectively) with some redundancies (23 in total) (**Figure 5C** and **Supplementary Figure S4**). The result revealed that overall B3/REM, bZIP, FHY/FAR, or NAC domain harboring TFs exhibited much stronger binding affinity, while ERF and C2H2 members showed relatively weaker binding affinity to the *pMYB98* fragments used in the assay (**Supplementary Figure S4**). The only TF that overlapped between the *in silico* prediction and Y1H-derived pools was ANTHOCYANINLESS2 (ANL2). Within the YIH bait constructs tested, ANL2 showed binding affinity only to the longest fragment that harbored *SaeM*, which was absent in the two shorter ones.

To further ascertain whether ANL2 was bound to the *SaeM*, we assessed the DNA affinity purification sequencing (DAP-seq) data available in the Plant Cistrome Database (O'Malley et al., 2016), which showed that one of the 11-bp-long ANL2-binding sites (DHATTWAWDRH) overlapped with the *SaeM* sequence with a 2 bp extension at the 3′-end of the *SaeM*. Additionally, the d-repeat had a 4 bp overlap to the DHATTWAWDRH motif at its 5′-end. The d-repeat mutation (ACACAT > GTGAGC) resulted in changes to the third and fourth conserved bases (AT) of the ANL2-recognition motif. When the wider functionally active region of pMYB98 was analyzed, it was observed that there are several ANL2 binding motifs within and near *SaeM* (**Figure 5D**).

The DAP-seq data additionally revealed that the ANL2 homolog HD TFs HOMEODOMAIN GLABROUS 1 (HDG1) and HDG7 recognize similar motif but at the reverse strand (DYHWTWAATDH). Additionally, the binding motif of yet additional HD member, HDG11 (also referred to as ENHANCED DROUGHT TOLERANCE 1; EDT1), was very similar to that of ANL2 as well (**Figure 5D**). The experimentally confirmed DNA-protein interactions database ePlant (Austin et al., 2016) showed that at least ANL2 and HDG1 do interact with *pMYB98*.

The *SaeM* and associated ANL2-recognition motifs appear to harbor single nucleotide polymorphisms (SNPs) as assessed among 1,135 Arabidopsis accessions retrieved from 1001 Genomes Project (Alonso-Blanco et al., 2016). Interestingly, however, the SNPs were localized specifically at the 6^th^ and 10^th^ positions of the ANL2-binding site, which could be tolerated for either ANL2, HDG1, HDG7, and ETD1 binding (O'Malley et al., 2016) (**Figure 5D**).

*ANL2* is expressed in diverse plant tissues (**Figure 5E**), and the reporter construct harboring its 1.5 kb promoter (*pANL2*) showed that it drives expression at least at SCs and CC of the FG (**Figure 5F**). However, the *anl2^-/-^* single T-DNA insertion mutant did not bring any discrepancy to the PT guidance, its reception, and overall sed-set, even though we observed sporadic ovules exhibiting *pMYB98*-driven *GFP* expression at all FG component cells (strongest at the SCs) in the *anl2^-/-^* mutant (**Supplementary Figure S5**).

Additional pocket of multiple ANL2/HDG1 binding motifs was located further downstream of *SaeM*, at −236 to −218 bp. Earlier observations made on the 5′- and 3′-deletion series strongly suggests for the active involvement of this *cis*-region in SC-specific expression as well as the shortening of the *pMYB98* from −350 to −194 bp in the 5′-deletion series and deletion of −251 to −121 bp fragment in the 3′-deletion series led to significant decrease in the GFP signal (**Figure 1** and **Supplementary Figure S1**). The putative ANL2-binding pocket falls within the overlapping region of these two deletion series (−251 to −194 bp).

### pMYB98 activation feature is conserved in Brassicaceae family members

Our earlier observation showed that the concatemers of red fragment, that harbors the *SaeM* and two ANL2-recognition motifs brings about reporter expression at the SCs and the SC-exclusivity increased with the increase in the number of its concatemers (**Figure 3B** and **Supplementary Figure S3**). Furthermore, we found that much higher number of Brassicaceae-derived *pMYB98*s harbor *SaeM* as compared with their non-Brassicaceae derived counterparts.

**Figure 6:**
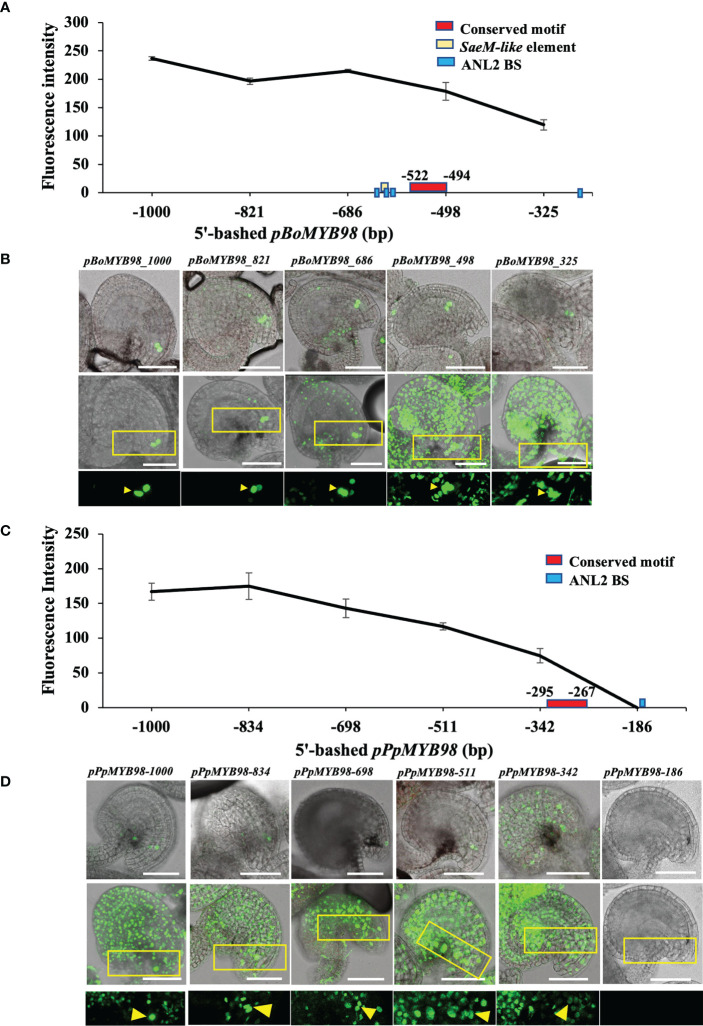
*SaeM-*dependent *MYB98* regulatory mechanism is conserved in Brassicaceae member. **(A, B)** Fluorescence intensities of 5′-deletion series of *Brassica oleracea*–derived *pMYB98* (*pBoMYB98*) reporter lines exhibited exclusive SC-specificity with its 1 kb promoter, which gradually waned in −821 and −686 bp reporter lines but was completely lost at reporter lines with −498 bp or shorter promoters (*n = 12*). The region −686 to −498 harbors *SaeM-like* elements along with multiple ANL2-binding sites (BS) and the MEME-derived conserved region. **(C, D)** Fluorescence intensities of 5′-deletion series of *Prunus persica*–derived *pMYB98* (*pPpMYB98*) reporter lines exhibiting non-exclusive SC-expression (*n = 12*). The promoter lacks *SaeM-like* element and harbors a single ANL2-binding site within 186 bp upstream of ATG. The MEME-derived weakly conserved motif falls within −342 to −186 bp region. The top rows of **(B, D)** show representative images used for fluorescence intensity measurement, the middle and bottom rows show the representative z-stack-merged (maximum projection) images and respective magnified regions. Distinguishable SCs are pointed with arrowheads. Scale bar = 50 μm.

To confirm whether such conservation is translated into *in vivo* expression of respective *MYB98*, we selected cabbage (*Brassica oleracea*; Brassicaceae) derived *MYB98* (*BoMYB98*; Bol029896) and peach (*Prunus persica*; Rosaceae) derived *MYB98* (*PpMYB98*; Prupe.5G172400) to assess 5′-promoter deletion reporter series in Arabidopsis. The two were selected based on their relatively distant and closer clustering with *pAtMYB98* (Kindly check **Figure 2A** and **Supplementary Figure S2**). While both promoters exhibited a gradual drop in reporter signal with decreasing promoter length, only *pBoMYB98* exhibited exclusive SC-specific expression with a 1 kb promoter fragment which was largely retained with −821 and −686 promoter fragments as well. However, the lines harboring −498 and −325 bp promoter fragments completely lost exclusive SC-specific expression. Both of the-498 and -325 bp *pBoMYB98* fragments completely lacked the region harboring *SaeM-like* element, multiple ANL2-binding sites, and the MEME-derived conserved site (**Figures 6A, B**). *pPpMYB98*, which lacked a *SaeM-like* element and harbored only one ANL2-recognition motif, failed to exhibit exclusively SC-specific expression even with its 1 kb promoter fragment (**Figures 6C, D**) supporting the finding that *SaeM* and multiple ANL2-binding sites are required for SC-specific expression in Arabidopsis. Furthermore, our earlier mutation and fragment combination observations showed that the TF binding within the *SaeM-like* element is more crucial than that at any other sites for exclusive SC-specific as well as overall reporter expression levels (**Figures 3**, **4**).

## Discussion

SC-specific expression of *MYB98* is quintessential for synergid fate determination and regulation of the PT guidance network (Kasahara et al., 2005; Punwani et al., 2008). In Arabidopsis, ectopic expression of *MYB98* leads to fate switch of other FG components (Zhang et al., 2020) while loss of MYB98 in SCs critically hinders PT guidance near the micropylar end of the FG (Kasahara et al., 2005).

Previous studies have shown that the expression of *MYB98* in the CC is actively repressed by a MADS box member AGL80, which binds to multiple CArG sites at *pMYB98*, one of which resides just downstream of the conserved region identified by MEME analysis in this study. Previous research has also shown that *MYB98* expression in the SC is affected by the TFs CCG and CBP1 expressed at CC potentially *via* cell-to-cell communication during the early stage of FG development (Li et al., 2015; Susaki et al., 2015). However, the mechanism behind is still unclear. Since we observed *ANL2* expression at both ECs and CC of a mature ovule, potential involvement of the gene in regulating the two cannot be disregarded. *pMYB98* additionally harbors a 7-bp-long “GTAACNT” element just downstream of the CArG box. The element has been characterized as the MYB98 recognition site itself (Punwani et al., 2008), suggesting the regulation of *MYB98* in a positive-feedback loop. Furthermore, a more recent study by Noble et al. (2022) reported an 8-bp-long SEEL element, “*TAATATCT*” in the *LORELEI* (*LRE*) promoter, deletion or mutation of which led to reduced expression of the gene in SCs. The element is recognized by MYB-related REVEILLE (RVE) TFs. Interestingly, available DAP-seq data indicate that *pMYB98* also harbors multiple *SEEL-like* elements within the −400 to −300 bp region. However, the sufficiency of the element in driving SC-specific expression itself is yet unclear. This study reveals additional mechanisms as to how *MYB98* expression is spatially regulated during FG development, showing that 84 bp fragment of the *MYB98* promoter can drive SC-specific expression and mutation within 16 bp *SaeM* completely masks its SC-specific expression potential.

### Synergid-specific activation element of MYB98 is crucial for dynamically driving exclusive SC-specific expression

We used a variety of reporter constructs and sequence analyses to conclude the 16-bp-long *SaeM*, “catttACACATtaaaa,” as a crucial *cis*-region behind the SC-specific expression of *MYB98*. Mutation within it caused the loss of otherwise precisely SC-specific expression driving potential of 169-bp-long BYRG fragment (**Figures 4B, C**) strongly suggesting that *SaeM* not only activates *MYB98* expression in SCs but is also involved in *MYB98* non-activation in non-SCs. It is further supported by the occurrence of *SaeM* in much greater proportion of the exclusively SC-specific gene promoters as compared with those expressed either in all or only in CC and EC of the FG (**Figure 5A**).

The *SaeM* and its sandwiching region contain several ANL2-recognition motifs. Comparison of *SaeM* with known plant *cis*-elements in PlantCare revealed its partial similarity to a previously characterized light-responsive element from *Pisum sativum rbcS-3A-gene* promoter, Box-III (ATCATTTTCACTATC) (Green et al., 1987). Interestingly, however, the BOX-III harbors mismatches at an essential *SaeM* region (as shown by the mutational analysis) (**Supplementary Figure S6A**). This suggests a different and unique function for *SaeM* as compared with BoxIII. Additionally, the 6-bp-long d-repeat just upstream of *SaeM* overlaps with the GCN4 motif, “TGAGTCA,” which had been characterized as an essential and sufficient *cis-*element for endosperm-specific expression of a rice glutelin gene through binding of the bZIP TF member, Opaque-2 (O2) (Wu et al., 1998). Our observations showed that exclusive SC-specific expression is not perturbed by the mutation of the “TGAGT” bases within the GCN4 motif. However, the expression potential of the *cis*-fragment was completely lost upon that in combination with the mutation of a similar sequence (d-repeat) within *SaeM*. The mutation of only the latter one, on the other hand, led to ovule-wide expression at sporophytic cells with no distinguishable SCs (**Figures 4B, C**), strongly suggesting for the non-exclusive expression potential of putative bZIP-like TF bound at GCN4 motif. Such observation is strongly supported by our additional observation that exclusivity in SC-specific expression was increased with the increase in the number of self concatemer of *SaeM* and two ANL2 binding motifs harboring 44-bp-long R fragment (**Figures 3B** and **Supplementary Figure S3**). Interestingly, however, we could not see any PT guidance defect in the *anl2^-/-^* mutants. More comprehensive study in the future with the double to quadruple mutant lines of *ANL2*, *HDG1*, *HDG7*, and *ETD1* may conclude whether the absence of PT guidance aberration in the *anl2^-/-^* mutant was due to the complementation effect of the *ANL2*-homologs.

### Synergid-specific activation element of MYB98–dependent regulatory system is conserved among Brassicaceae members

Our study showed that the conservation of *SaeM* in greater proportion of Brassicaceae-derived *pMYB98* is translated to its involvement in driving SC-specific expression of the associated gene in Brassicaceae members. We confirmed the case *via* 5′-deletion series of *B. oleraceae*– and *P. persica*–derived *pMYB98*s. The former harboring *SaeM* exhibited largely SC-specific reporter expression unless the region harboring the *SaeM* element was deleted. The latter lacking the element often exhibited the reporter expression non-exclusively even at its full length (1 kb) (**Figure 6**). However, it is important to note that the *B. oleraceae*–derived *SaeM-like* element harbors few base dissimilarities to that of Arabidopsis. One of the mismatches can seriously hinder the potential binding of ANL2 and its homologs to the element, even though the putative binding sites at its up- and downstream are intact (**Supplementary Figure S6B**). However, as the 5′-deletion series showed, it can be tolerated for driving SC-specific *BoMYB98* expression. Furthermore, the element showed a much higher proportion of conservation among the SC-specific gene pool of Arabidopsis (**Figure 5B**). In addition to the Brassicaceae members, phylogenetic relatedness roughly suggested the conservation of *SaeM*-dependent regulatory features in Malvaceae members as well (**Figure 2A** and **Supplementary Figure S2**). Such family-wide conservation of regulatory feature is not uncommon. An earlier study on AGL80, a MADS-box protein, also showed its Brassicaceae family-wide conserved function of *MYB98* repression at CC (Zhang et al., 2020). Apart from Brassicaceae members, a rice *MYB98* (*OsMYB98*) also exhibited synergid-specific expression. Interestingly, its promoter also harbors *SaeM-like* element at −980 to −965, which shows greater similarity at its 3′-end and also constitutes a putative ANL2 recognition motif (**Supplementary Figure S6C**). It is plausible yet unconfirmed whether the element is part of SC-specific *OsMYB98* expression as well.

## Conclusion and perspective

After nearly two decades of the discovery of SC-specific *MYB98* and its quintessential role in PT guidance, the current study has discovered a novel *cis*-regulatory element, *SaeM* (catttACACATtaaaa), is dynamically involved in the activation of the *pMYB98* in SCs while coordinating its effective non-activation at non-SCs. We have demonstrated that an 84-bp-long *cis*-region harboring the *SaeM* is sufficient to drive SC-specific expression in Arabidopsis. Interestingly, the *SaeM*-dependent regulatory mechanism was found highly conserved among Brassicaceae members. The element constitutes a putative ANL2 and homolog recognition motif.

**Figure 7:**
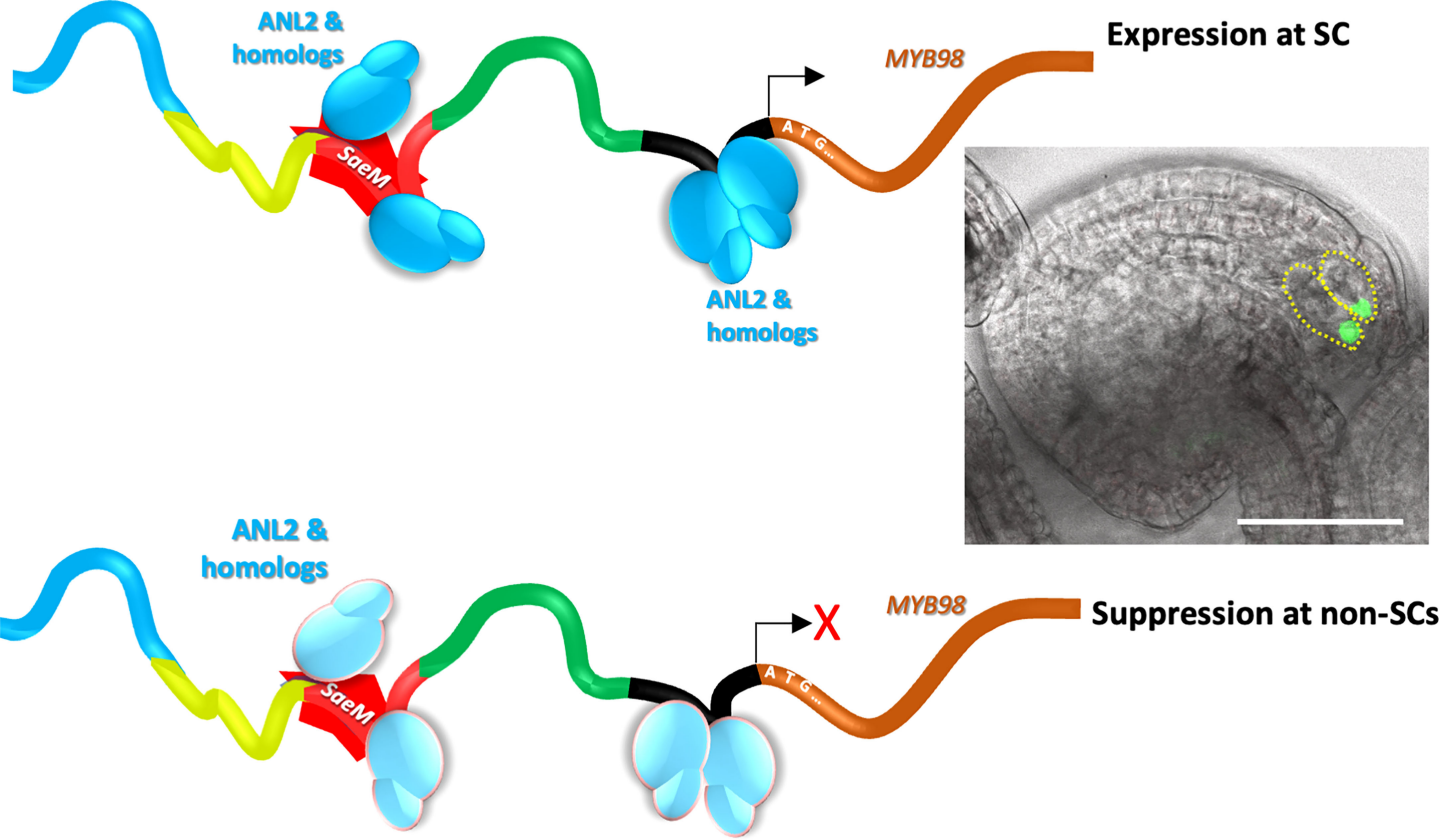
A model proposed for the *SaeM*-dependent synergid-specific expression of *pMYB98*. The *SaeM* activates *pMYB98* in synergid cells, which is potentially mediated by ANL2 and/or homologs bound to it. The process is further enhanced by the putative ANL2-binding pocket located further downstream. At non-synergid cells, however, the *SaeM* remains ineffective in driving MYB98 expression either due to potential binding of yet unknown repressor or the ANL2 and/or homologs bound to it actively contribute to the repression process.

Our observations suggest for two possible reasons behind the *SaeM*-dependent dynamic regulation of *MYB98*. Simply, it is possible that the element is selectively bound by activator at the SCs, which is impaired or replaced by the repressor at non-SCs. In other case, the ANL2 and/or homologs bound to the *SaeM* may function as activator at SCs and as active repressor at non-SCs (**Figure 7**). It is plausible but how the ANL2 and/or homologs would confer such dual functionality is yet unclear. A study by Zhang et al. (2020) showed that an EAR motif harboring transcription factor, AGL80 in association with the EAR-motif bound TPL/SAP18 co-repressor actively represses *MYB98* expression in CC. While some of the ANL2-like TFs too harbor putative EAR-motifs (**Supplementary Figure S7**), it is yet unclear whether they play any role in *SaeM*-dependent dynamic regulation of *MYB98*. A more comprehensive study on if and how ANL2 and homologs are involved in the dynamic regulatory function of *SaeM* is warranted.

## Materials and methods

### Bioinformatics

#### *MYB98* homolog retrieval and assessment

Putative *MYB98* homologs were screened from the phytozome database (https://phytozome-next.jgi.doe.gov/phytozome/) using the “MYB98” keyword to retrieve respective amino acid and 1 kb 5-upstream sequences using BioMart tool (341 sequences retrieved). The sequences has been referred to as “promoters” elsewhere in the manuscript. Additionally, BLASTp was carried out against the Phytozome amino acid database using AtMYB98 as a query sequence with default parameters. Among the top 100 sequences retrieved, all but two were annotated as “MYB98.” Interestingly, unlike Arabidopsis, several species, including those of Brassicaceae members, harbor more than one *MYB98* homologs. A total of 107 sequences were retained in the keyword-derived sequence pool after selecting a single *MYB98* homolog per species with lesser *e*-value with longer alignment lengths and after removing those with the promoter of < 1 kb length or ambiguous sequences. It constituted all MYB98 blastp derived sequences except for one which was annotated as *MYB119*.

To further ascertain the true MYB98 homologs, amino acid sequences of all *Arabidopsis*-derived R2R3 MYBs (129 in total) (Stracke et al., 2001) were aligned with the Phytozome-derived unique sequences (236 in total; with amino acid identity values < 95%) followed by their maximum-likelihood phylogeny using Jalview (Waterhouse et al., 2009), PhyML (Guindon et al., 2010), and iTOL (Letunic and Bork, 2021). Interestingly, not all of the putative MYB98 homologs clustered with Arabidopsis MYB98. Hence, we took the members clustered with the AtMYB subgroup 25 (SG25) members (AtMYB64, AtMYB98, and AtMYB119) as putative MYB98 homologs. A separate analysis was carried out taking the MYB98 homologs and AtMYB SG25 members (**Supplementary Figure S2**). The promoter sequences of 50 *MYB98* homologs along with *AtMYB98*, *AtMYB64*, and *AtMYB119* were taken for native motif search using MEME-Suite (https://meme-suite.org/meme/tools/meme) (Bailey et al., 2009).

#### TF and associated *pMYB98 cis*-element prediction

PlantRegMap (Tian et al., 2019) was used for the prediction of the TFs potentially binding to the 5′-deletion series derived functionally active 191 bp region with default parameters (threshold *p*-value ≤ 1e-4). ANL2 and its three homologs were aligned using MUSCLE in Jalview (Waterhouse et al., 2009) followed by the curation in Bioedit. Conserved domains and active residues in each of them were determined *via* CD search (https://www.ncbi.nlm.nih.gov/Structure/cdd/wrpsb.cgi).

#### SNP data retrieval and assessment

For the assessment of SNPs within the 1 kb region of *pMYB98*, the data of 1135 Arabidopsis accessions was retrieved from the Arabidopsis 1001 Genomes Project (Alonso-Blanco et al., 2016). The sequence Col-0–derived 1kb *pAtMYB98* (upstream of translation start site) was manually aligned with the database derived sequences, and the SNP sites were determined. Associated SNP graph was plotted and specific SNP types at the site of interest (*SaeM* and ANL-binding sites) were analyzed.

#### Motif alignment

The *SaeM* element (CATTTACACATTAAAA) was taken for their independent alignment against the 1 kb promoters of the genes from various gene pools (FG component cell specific, pollen specific, non-specific, etc.) Song et al. (2020) using the motif alignment program MAST (http://www.meme.sdsc.edu/) (Bailey and Gribskov, 1998) with default parameters (*e*-value < 10, *p*-value < 0.0001). The exclusive SC-specific expression of a gene was considered true when it had null expression value at all replications of CC and EC but > 0 values at those of SCs.

### Plant materials

Arabidopsis Col-0 was used as the background line (WT) as well as for transformation purposes in the study. The plants were grown at *in vivo* conditions in the culture room with 24/8 hr (light/dark) photoperiod and ~25°C temperature at Fujian Agricultural and Forestry University, Fuzhou, China.

### Cloning and vector construction

Most of the constructs were prepared using In-Fusion HD Cloning Kit (Takara, 121416) following the manufacturer’s protocol and a few were prepared *via* restriction digestion cloning procedure using the FastDigest enzymes (Thermo Fisher Scientific).

#### 5′-promoter bashing

For the 5′-promoter deletion series, DNA was extracted from WT *Arabidopsis thaliana*, *Brassica oleracea*, and *Prunus persica* following the CTAB extraction method (Clarke, 2009), and desired lengths of 5′-deleted promoter fragments upstream of ATG was amplified using respective primers. The lengths of each deletion series fragment have been shown in the associated figures. pCAMBIA1305 (PC1305) plasmid was used as a base vector for deletion series constructs preparation in such a way that each promoter fragment is cloned upstream of the reporter (*GFP* for *A. thaliana* during the initial assessment, and *H2B-GFP* for *B. oleracea* and *P. persica* to assess cross-species feature conservation). Associated primers have been provided in **Supplementary Table S8**.

#### Addition/deletion of pMYB98 cis-fragments

169 bp functionally active *pMYB98* region derived from the 5′/3′-deletion series in combination with the putative TF binding site prediction and the sequence region of MEME-derived putatively conserved motif lying within the functionally active *pMYB98* region (23 bp sequence at 5′-region of 29 bp long motif) were targeted for the assessment of their role in reporter expression.

As an SC-specific positive control, the 1.5 kb of *pMYB98* was amplified using the pMYB98_1.5k.fwd and pMYB98_1.5k.rev primers and cloned into the H2B-GFP reporter construct. Its functionally active 169 bp region lacking version (*pΔMYB98*) was prepared by amplifying the first half with pMYB98_1.5k.fwd and pMYB98_BYRGdel.rev (855 bp) and the latter half with the pMYB98_BYRGdel.fwd and pMYB98_1.5k.rev (543 bp) followed by their assembly and incorporation upstream of the *H2B-GFP* of the reporter construct using the In-Fusion HD Cloning Kit (Takara, 121416) following the manufacturer’s protocol. For the preparation of the conserved region lacking version of the promoter (*pδMYB98*), the reverse primer of the first half and forward primer of the second were respectively substituted with MYB98proDelcon_infu.rev and pMYB98_Delcon.fwd while using them as they were and assembled in the reporter construct as mentioned earlier. The amplified promoter fragments were assembled and incorporated into the reporter construct following the aforementioned procedure.

For the inclusion effect of the aforementioned region on restricting the reporter expression at SCs, we amplified 346-bp-long *pCaMV* along with an additional 238 bp sequence at its upstream and 15 bp at its downstream (599 bp in total) from the pC1302 plasmid using pCaMV.fwd and pCaMV.rev primers. The amplicon was incorporated upstream of *H2B-GFP*, and the prepared construct was used as a negative control for SC-specific expression. The functionally active 169 bp fragment and the MEME-derived conserved motif harboring regions were independently incorporated at −227 bp of *pCaMV* (−239 bp from ATG) to construct *pΔCaMV* and *pδCaMV*, respectively. For the preparation of *pΔCaMV*, the first half of *pCaMV* was amplified from pCAMBIA1302 using pCaMV.fwd and pCaMVpt1A.rev primers (409 bp), and the second half was amplified using the pCaMVpt2.fwd and pCaMV.rev primers (239 bp). For the *pδCaMV*, only the reverse primer of the first half of *pCaMV* was substituted with pCaMVpt1B.rev while others were used as they were. The 169 bp fragment for *pΔCaMV* was amplified using pCV-A.fwd and AB-pCV.rev, and the MEME-derived conserved region harboring fragment for *pδCaMV* was amplified by substituting the forward primer with pCV-A.fwd. Respective constructs were prepared *via* infusion cloning as mentioned above. Primer information is provided in **Supplementary Table S8**.

#### *pMYB98* sub-fragment combination and fragment mutation

For the combination of the B, Y, R, and G sub-fragments within the functionally active 169 bp region, each sub-fragment was amplified *via* primer extension of respective 5′ region annealing forward and reverse primers harboring 3′-sequence variation based on target fragment combinations. For the self concatemers of each fragment, the whole target fragment was synthesized. For the preparation of putative repeat sequence mutation, the target region was amplified using the mutation-harboring primers followed by the sub-fragment assembly. Each sub-fragment combination or mutated fragment was incorporated upstream of *CaMV* minimal promoter (*MP*) of the *H2B-GFP* reporter construct. (Primer information can be provided upon request).

#### Bait constructs preparation for Y1H

Three *pMYB98* bait fragments of 40 bp (−679 to −640 bp), 60 bp (−679 to −620 bp), and 139 bp (−679 to −541 bp) were amplified using pMYB98-pHISi_fw (P1) as forward primer and respective reverse primers of pMYB98-pHISi40bp_rev, pMYB98-pHISi60bp_rev, and pMYB98-pHISi139bp_rev. Each amplification product was cloned into the pHISi yeast expression vector independently.

#### Preparation of *pANL2* reporter construct

For the preparation of *pANL2::H2B-GFP* construct, the 1.5 kb promoter region upstream of ATG was amplified using pANL2_1.5k.fwd and pANL2_1.5k.rev primers (**Supplementary Table S8**) and cloned into the *H2B-GFP* reporter construct *via* infusion cloning.

### Plant transformation and assessment

Transgenic seeds were derived from the Col-0 Arabidopsis plants *via* the floral dip transformation method following the protocols described by Bent (2006). Simply, the acetosyringone-treated and Silwett-supplemented *Agrobacterium* transformation solution (OD ~ 0.8; prepared after the single colony selection followed by the culture and sub-culture steps) of intended constructs were used for the transformation. The culture room-grown plants with numerous floral buds were taken and the opened flowers were removed before dipping the buds in the *Agrobacterium* solution. The plants were dipped in the fresh transformation solution every other day three consecutive times and left for seed set. The seeds were collected from the mature silique and plated in the selection antibiotics supplemented MS media. *MYB98* being FG-specific, we took the plants harboring at least ~50% of the ovules with the distinguishable SC-specific GFP markers for the fluorescence intensity assessment. Nine to 20 independent transgenic plants per each construct were used for the observation in the study.

### Reverse transcription polymerase chain reaction

Tissues from five different plant parts, that is, flower (stages 1-2, 3, 4-5, and 6-12) silique (very young, young, medium, and mature stage), leaves (young and mature stage), stem (young, medium, and mature stage), and root (upper part without root tips and lower part with root tips) were collected from Col-0Arabidopsis plants. RNA was extracted from the collected samples using RNeasy Plant Mini Kit (Cat no. 74904; Qiagen Shanghai, China), and cDNA was synthesized for the tissue-specific expression assessment of *ANL2* along with *Actin2*. Traditional PCR machine (GeneAmp PCR System 9700 Thermo Fisher Scientific, Hongkong) was used for RT-PCR using Novogene 2x Taq Plus PCR mixture under the following condition: One cycle of pre-heating at 95°C for 5 min followed by 30 cycles of (95°C for 20 s, 59°C for 35 min, and 72°C for 1 min) and one cycle of (72°C for 7 min and 4°C for 10 min). Associated primer information has been provided in **Supplementary Table S8**.

### Confocal microscopy

Flowers at the stage of 12c (Christensen et al., 1997) were emasculated and microscopic observations were made after 24h. The siliques were excised, put on the observation slides, and the valves were removed using a syringe needle. The samples were covered with coverslip after adding a few drops of water and observation of the exposed ovules were made under MRC 1000 confocal laser scanning microscope (CLSM) (Bio-Rad) *via* the detection of GFP emission within the 500–530 nm wavelength range after excitation of the sample with an argon laser at 488 nm wavelength. A similar sample preparation procedure was followed for the sample observation under an epifluorescence microscope (Ex546/10/DM 565 LP/Em 590 LP) using a GFP filter. The collected images were analyzed using Leica LAS-X Life Science Microscope Software, Zen blue 2.3, and Fiji. The distinguishability of the SCs was determined in the 3D z-stacked images based on the position-specific (micropylar) typical twin-like nuclear localizations. The fluorescence images and respective numerical values were retrieved after the z-stack merges (maximum projection) unless mentioned otherwise (https://imagej.net/software/fiji/).

### Yeast-one-hybrid assay

The Y187 yeast strain was transformed with the three bait constructs independently and Y1H screening was carried out using the Arabidopsis TF library containing ~1,400 transcription factors following the protocol described by Mitsuda et al. (2010). Three *pMYB98* bait fragments of 40 bp (−679 to −640 bp), 60 bp (−679 to −620 bp), and 139 bp (−679 to −541 bp) were cloned into pHISi using the In-Fusion HD Cloning Kit (Takara, 121416) following the manufacturer’s protocol. Each bait vector was linearized by ApaI and transformed into the yeast strain YM4271 using PEG/LiAC method. Appropriate 3-AT concentration was selected after testing the bait vector harboring yeast cells for HIS3 reporter background expression. The yeast mating method derived haploid yeast cells were selected on screening plates (SD-Ura-His-Leu plus optimum 3-AT) and positive colonies were sequenced afterward.

### Pollen tube elongation assessment

The flowers at stage of 12c from target plants were emasculated and hand-pollinated with the pollens of the desired plant after ~40h after emasculation (Faure et al., 2002). The pistils were excised at defined hours after pollination (after 12h–24h) and fixed in FAA (10% formaldehyde, 5% acetic acid, and 50% ethyl alcohol). The fixed pistil tissues were softened in 1M NaOH for 4h and stained with aniline blue to visualize PTs in the pistil squashes (Jones et al., 2018) under CLSM or epifluorescence microscopes at 12h, 16h, and 24h after pollination (HAP).

### Statistics

Samples of all relevant experiments were collected at least in triplicates. Sample numbers have been provided with the associated figures. The collected numerical data were analyzed *via* Duncan’s multiple range test (DMRT) or Student’s t-test.

## Data availability statement

The datasets presented in this study can be found in online repositories. The names of the repository/repositories and accession number(s) can be found in the article/**Supplementary Material**.

## Author contributions

RK conceived the idea. RK and PA designed the experiments. RK and LX carried out the preliminary 3’- and 5’- deletion series experiments. LB and BP contributed to initial sequence assessments. NM carried out the Y1H experiment. PA guided and carried out the majority of the remaining experiments along with SZ. XL, CH, XW and JH assisted on phenotypic data retrieval. PA analyzed and curated final data and figures, and prepared the manuscript. LB, RK, BP, NM and SN contributed on the manuscript revision. All authors contributed to the article and approved the submitted version.

## Supplementary material

Supplementary Figure 1 Preliminary 3′-bashing of *pMYB98* indicates that deletion of either of the two *cis*-regions within the *pMYB98* drastically reduces the reporter signal. **(A)** Relative GFP-signal driven by 3′-deletion *pMYB98* series. The intact and deleted promoter segments have been denoted with the solid and dashed lines, respectively. Each of the deletion series was fused with the minimal promoter (*MP*) at its 3′-end (just upstream of *GFP*). The pairs of subsequent intact and deletion fragments exhibiting the drastic drops in GFP intensity (from −615 to −487 bp and from −251 to −121 bp) have been highlighted with the red background. +++ and - represent for the highest and no GFP intensities respectively. **(B)** Conclusion derived from the 3′-deletion promoter series **(A)** showing two distinct pockets deletion of which brought significant drops in the GFP signal.

Supplementary Figure 2 Maximum-likelihood phylogeny of the phytozome derived putative MYB98s and AtMYB subgroup 25 members. The subtree used for is highlighted in faint-red. The tree was prepared using Jalview (Waterhouse et al., 2009), PhyML (Guindon et al., 2010), and iTOL (Letunic and Bork, 2021).

Supplementary Figure 3
*R3-MP* driven *H2B-GFP* expression at all FG component cells with the strongest signal at synergids.

Supplementary Figure 4 Yeast-one-hybrid (Y1H) assay-derived transcription factors (TFs) exhibiting binding affinity to *pMYB98*. Three independent Y1H assays with the overlapping *pMYB98* sequences of 40 **(A)**, 60 **(B)**, and 139 bp **(C)** identified 23 unique TFs among which the three common TFs in all assays have been underlined. The domain family of each TF is shown in the parenthesis. The TFs exhibiting strong, weak, and weakest binding affinities are in red, blue, and light blue text. The TF (ANL2) overlapped with the *in silico* prediction has been denoted with an asterisk (*).

Supplementary Figure 5 Few ovules of defective *anl2^-/-^* lines exhibited *pMYB98* driven H2B-GFP expression at all FG component cells, which did not bring any changes to the pollen tube guidance and its ovular reception. (A) GFP signals at all FG component cells of *pMYB98* reporter harboring *anl2^-/-^* ovule with strongest signal at synergids. **(B–E)** Representative images of the reciprocal crosses between WT and *anl2^-^/-* at 24 HAP. **(F)** Pollen tube reception rate at different time points (each bar represents mean ± SE; *n* = 20).

Supplementary Figure 6
*SaeM* (boxed in green) comparison with known motif (BoxIII) **(A)**, similar region from *Brassica oleracea MYB98* promoter (*pBoMYB98*) **(B)**, and the *SaeM-like* element from rice *MYB98* promoter (*pOsMYB98*) **(C)**. *SaeM* and *SaeM-like* elements are within green boxes, mutation susceptible d-repeat within *SaeM* is in bold, putative ANL2-like TF-binding sites are in pink and shaded, and red arrowhead points to the potential mutation susceptible mismatch within *pBoMYB98 SaeM-like* element.

Supplementary Figure 7 Structure and conserved motifs assessment in ANL2 and homologs. **(A)** Schematic representation showing that ANL2 and HDG1 harbor two EAR motifs in addition to the HD- and START-domains, while HDG7 and ETD1 lack EAR motif. **(B)** Alignment of ANL2 and homologs with respective conserved motifs are annotated. Green oval circles mark the residues involved in DNA binding and red oval circles mark the residues involved in lipid binding.
