# Supplementary figures and images for: Discovery of a cis-regulatory element SaeM involved in dynamic regulation of synergid-specific MYB98
Source: Front Plant Sci. 2023 May 8;14:1177058. doi: 10.3389/fpls.2023.1177058 (PMC10200956; doi:10.3389/fpls.2023.1177058)

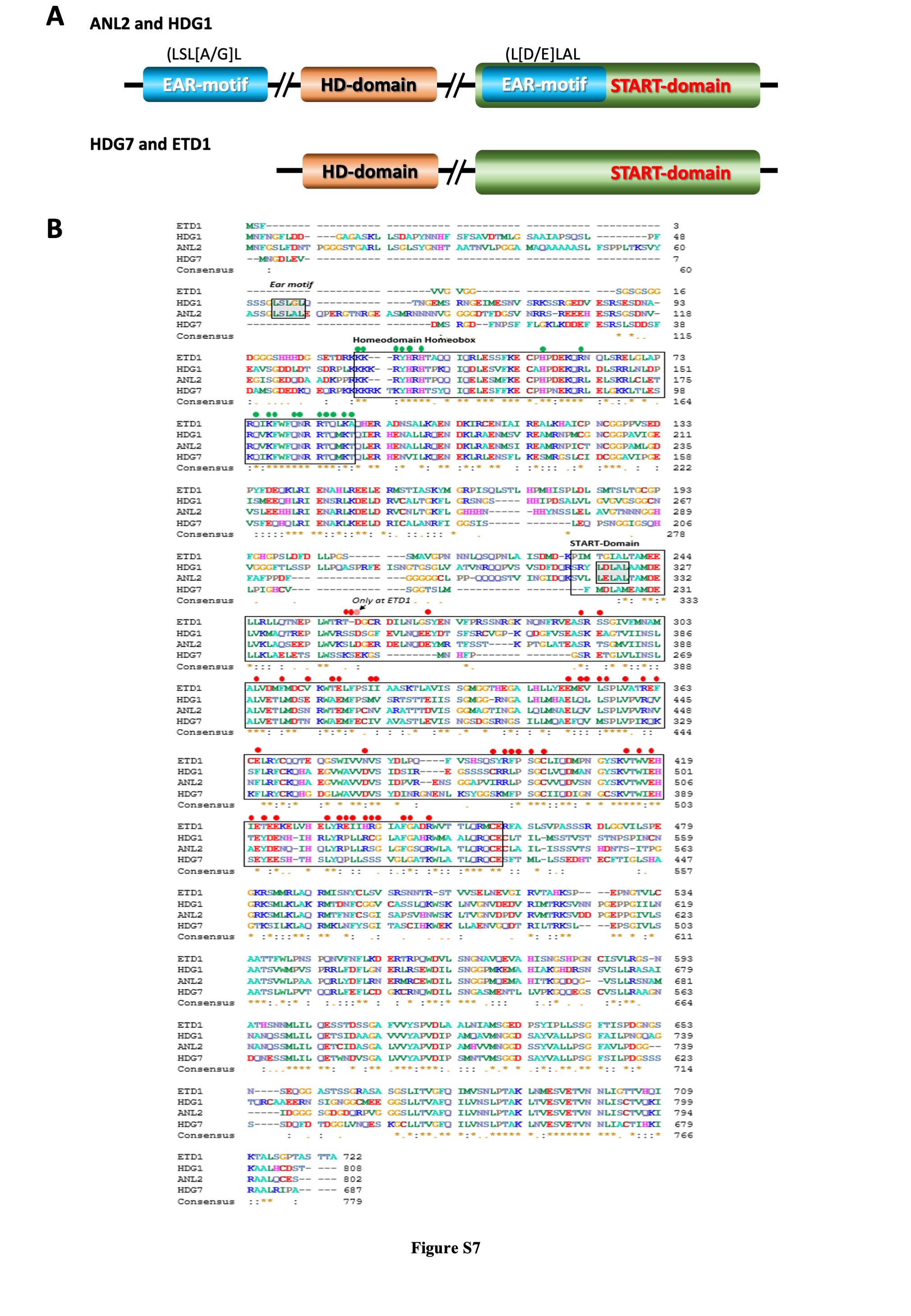

Supplement: Supplementary Figure 1 — Preliminary 3′-bashing of pMYB98 indicates that deletion of either of the two cis-regions within the pMYB98 drastically reduces the reporter signal. (A) Relative GFP-signal driven by 3′-deletion pMYB98 series. The intact and deleted promoter segments have been denoted with the solid and dashed lines, respectively. Each of the deletion series was fused with the minimal promoter (MP) at its 3′-end (just upstream of GFP). The pairs of subsequent intact and deletion fragments exhibiting the drastic drops in GFP intensity (from −615 to −487 bp and from −251 to −121 bp) have been highlighted with the red background. +++ and - represent for the highest and no GFP intensities respectively. (B) Conclusion derived from the 3′-deletion promoter series (A) showing two distinct pockets deletion of which brought significant drops in the GFP signal. [file Image_1.tiff]

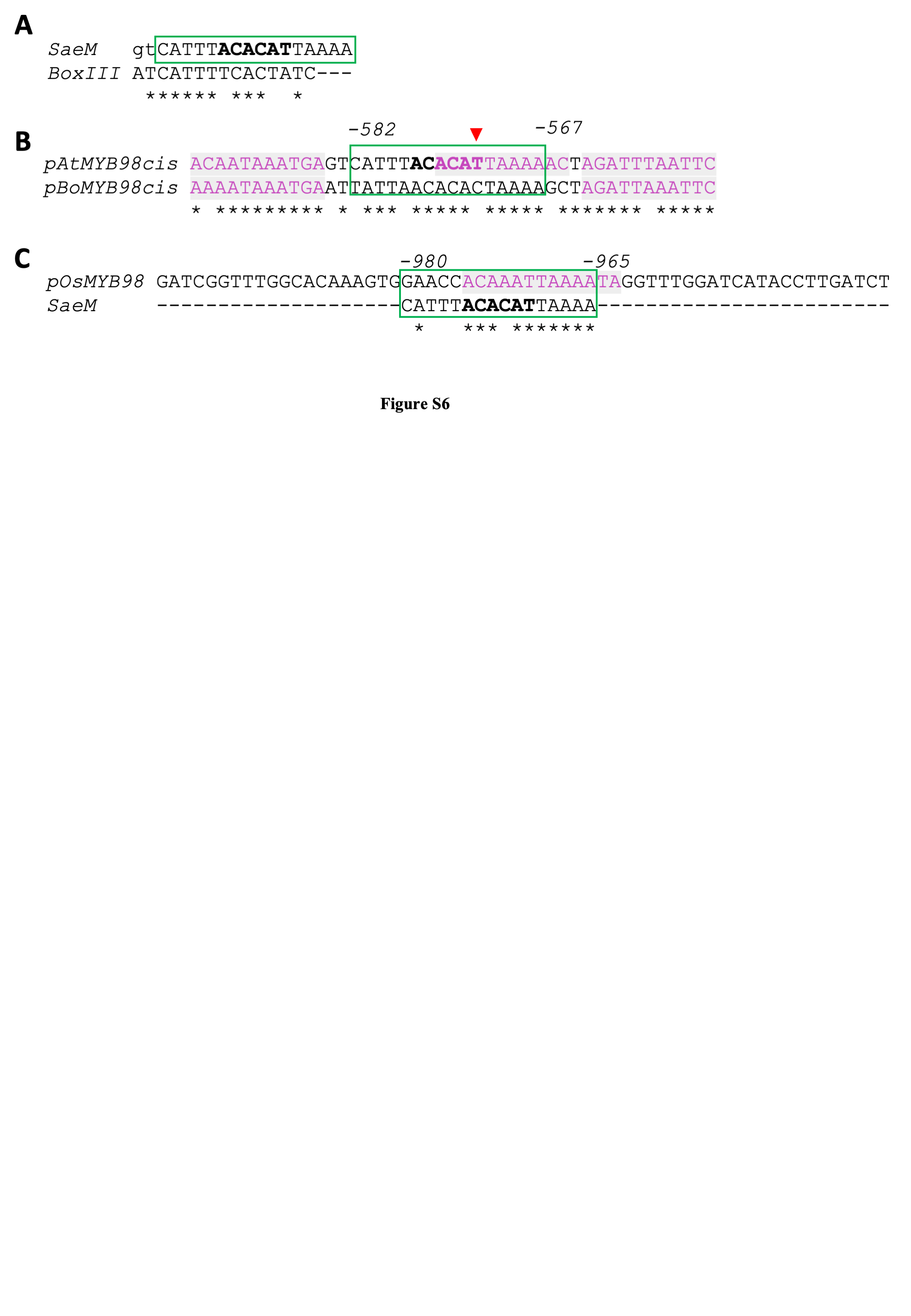

Supplement: Supplementary Figure 2 — Maximum-likelihood phylogeny of the phytozome derived putative MYB98s and AtMYB subgroup 25 members. The subtree used for is highlighted in faint-red. The tree was prepared using Jalview (Waterhouse et al., 2009), PhyML (Guindon et al., 2010), and iTOL (Letunic and Bork, 2021). [file Image_2.tiff]

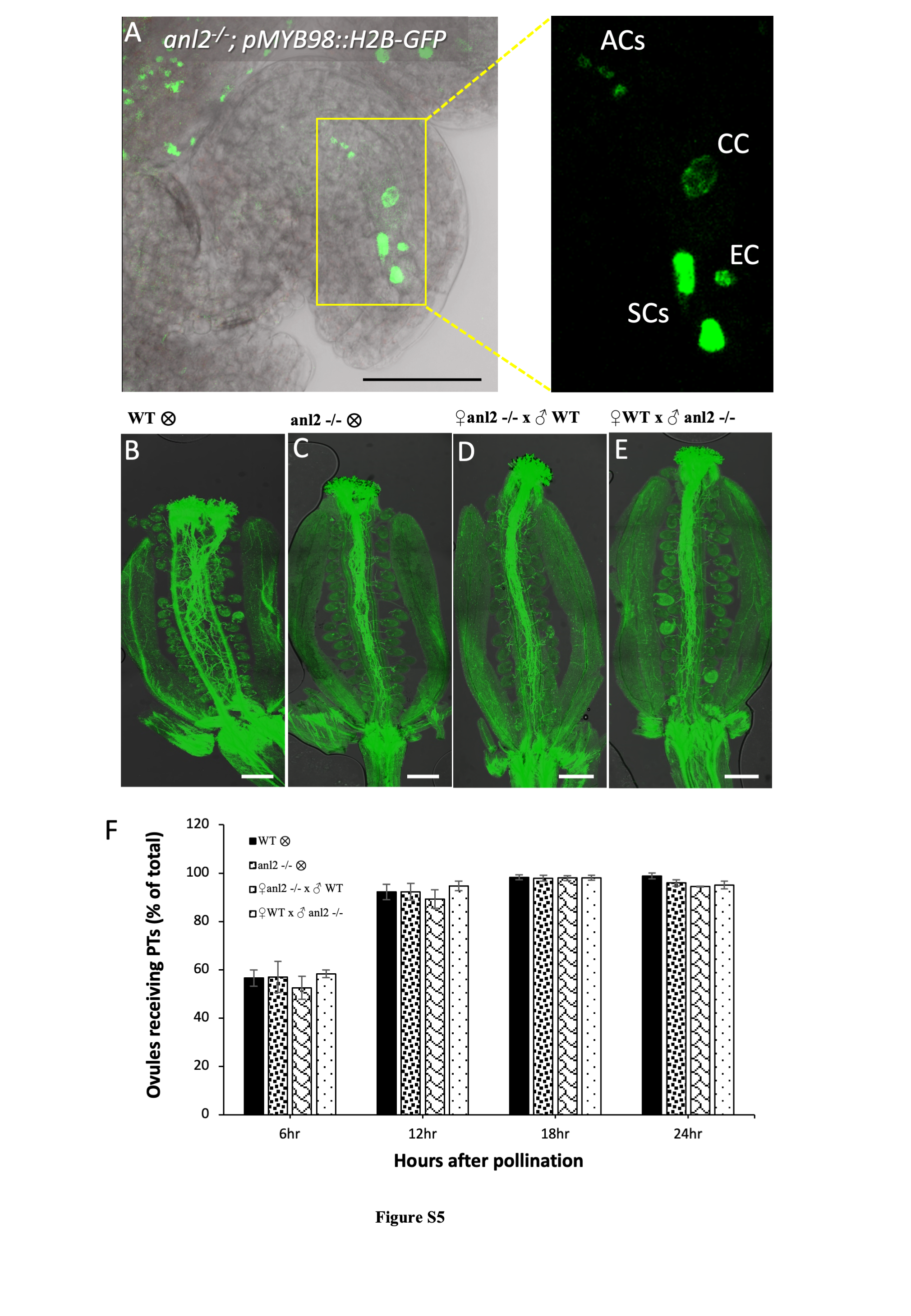

Supplement: Supplementary Figure 3 — R3-MP driven H2B-GFP expression at all FG component cells with the strongest signal at synergids. [file Image_3.tiff]

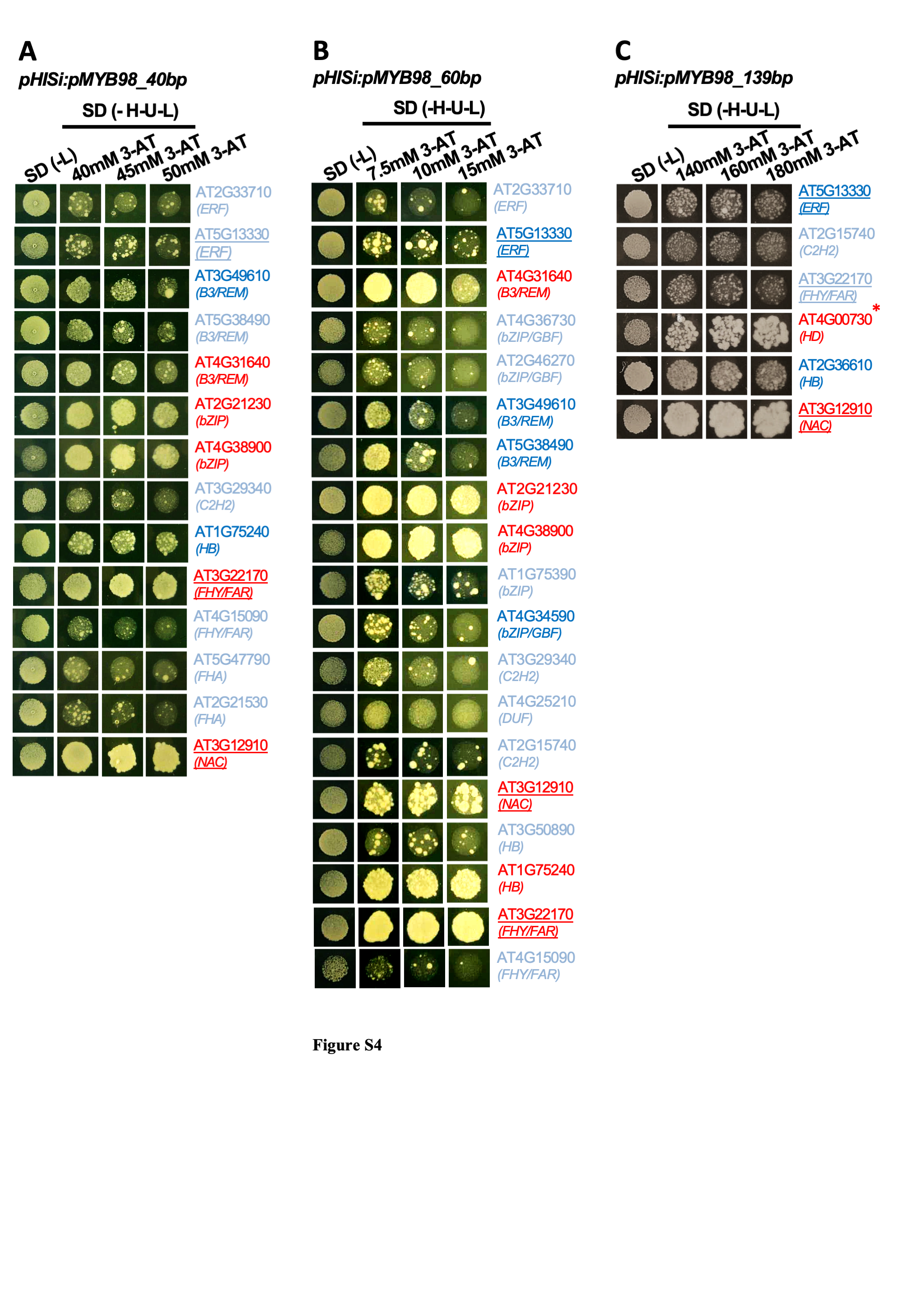

Supplement: Supplementary Figure 4 — Yeast-one-hybrid (Y1H) assay-derived transcription factors (TFs) exhibiting binding affinity to pMYB98. Three independent Y1H assays with the overlapping pMYB98 sequences of 40 (A), 60 (B), and 139 bp (C) identified 23 unique TFs among which the three common TFs in all assays have been underlined. The domain family of each TF is shown in the parenthesis. The TFs exhibiting strong, weak, and weakest binding affinities are in red, blue, and light blue text. The TF (ANL2) overlapped with the in silico prediction has been denoted with an asterisk (*). [file Image_4.tiff]

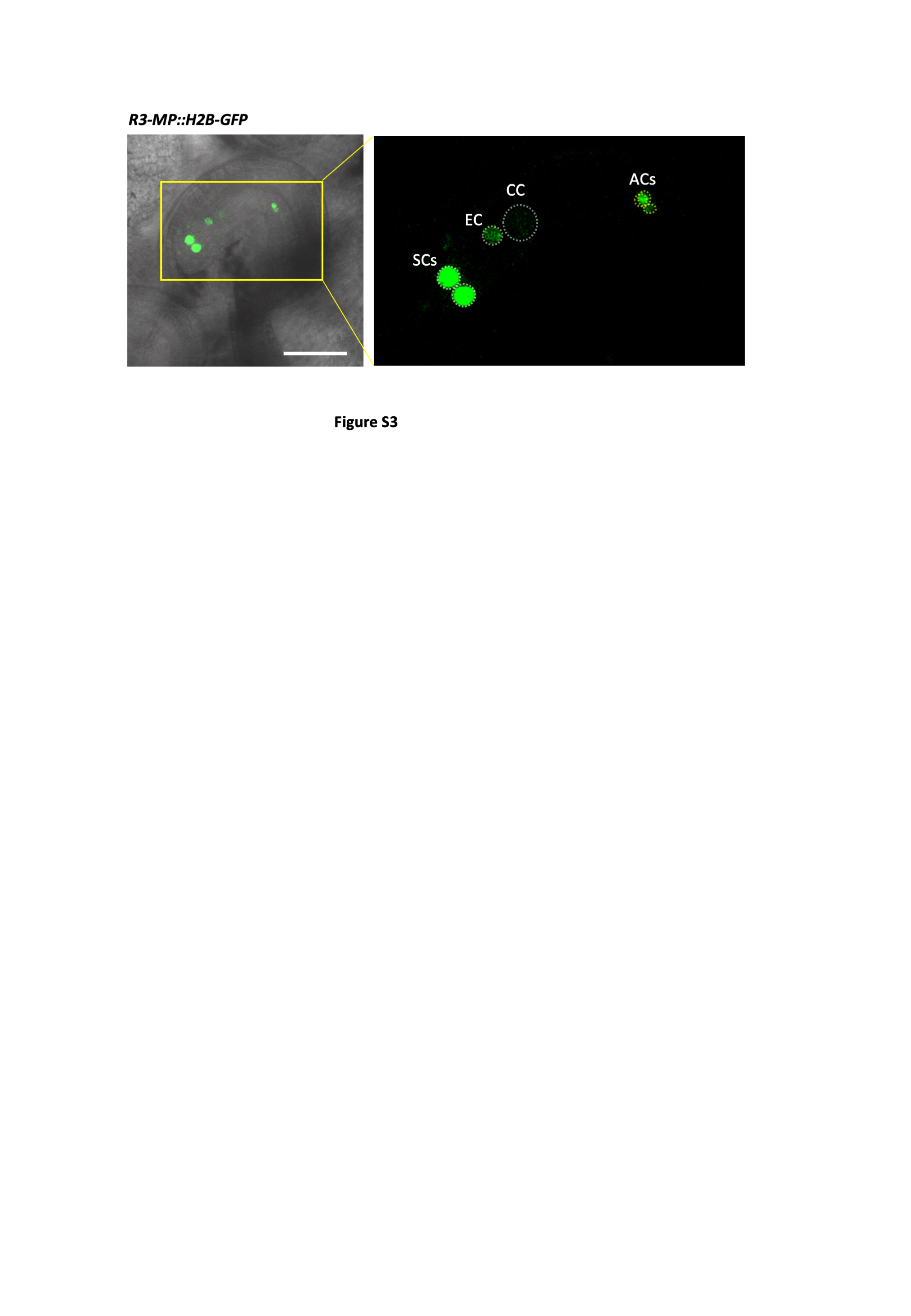

Supplement: Supplementary Figure 5 — Few ovules of defective anl2-/- lines exhibited pMYB98 driven H2B-GFP expression at all FG component cells, which did not bring any changes to the pollen tube guidance and its ovular reception. (A) GFP signals at all FG component cells of pMYB98 reporter harboring anl2-/- ovule with strongest signal at synergids. (B–E) Representative images of the reciprocal crosses between WT and anl2-/- at 24 HAP. (F) Pollen tube reception rate at different time points (each bar represents mean ± SE; n = 20). [file Image_5.tiff]

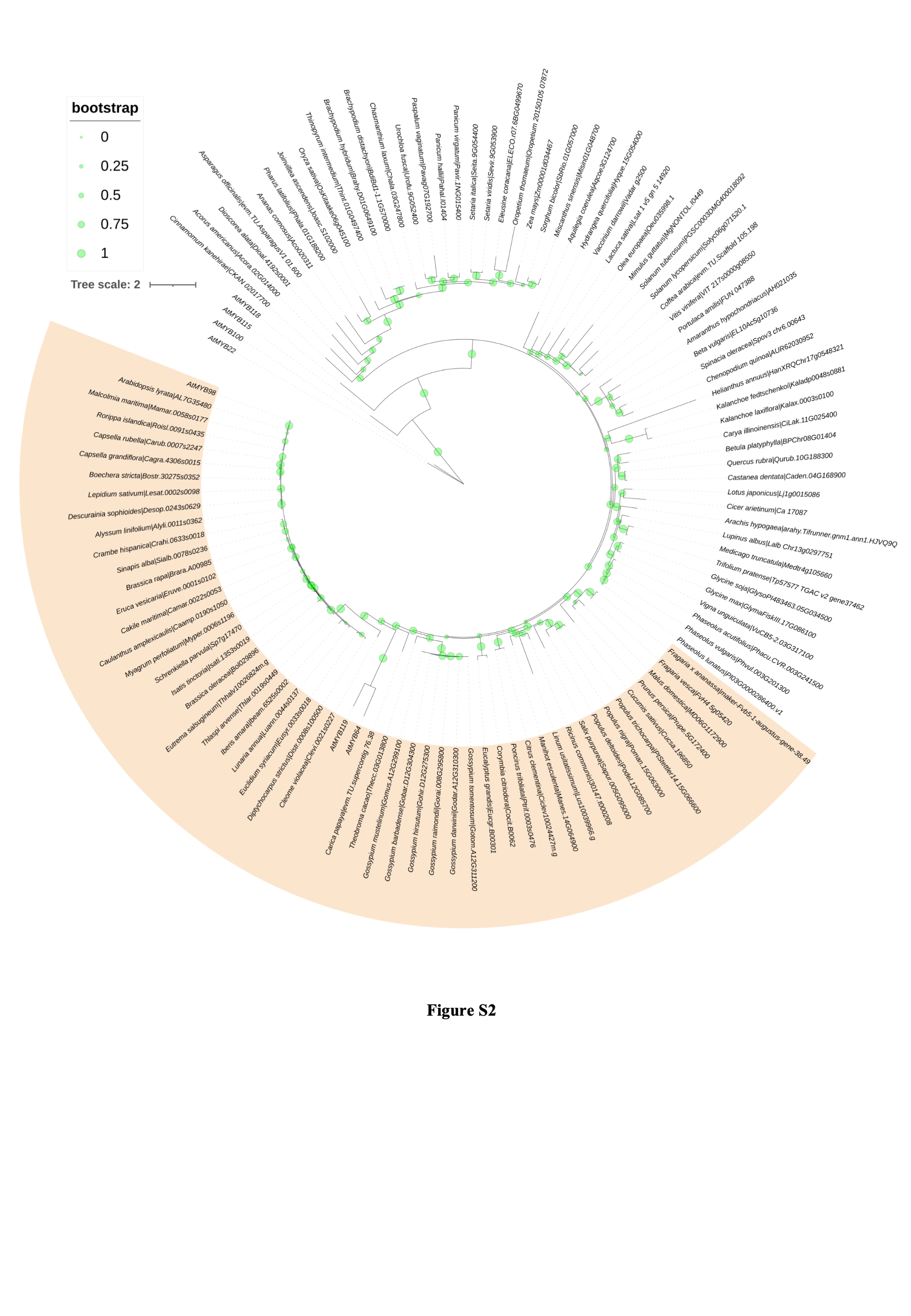

Supplement: Supplementary Figure 6 — SaeM (boxed in green) comparison with known motif (BoxIII) (A), similar region from Brassica oleracea MYB98 promoter (pBoMYB98) (B), and the SaeM-like element from rice MYB98 promoter (pOsMYB98) (C). SaeM and SaeM-like elements are within green boxes, mutation susceptible d-repeat within SaeM is in bold, putative ANL2-like TF-binding sites are in pink and shaded, and red arrowhead points to the potential mutation susceptible mismatch within pBoMYB98 SaeM-like element. [file Image_6.tiff]

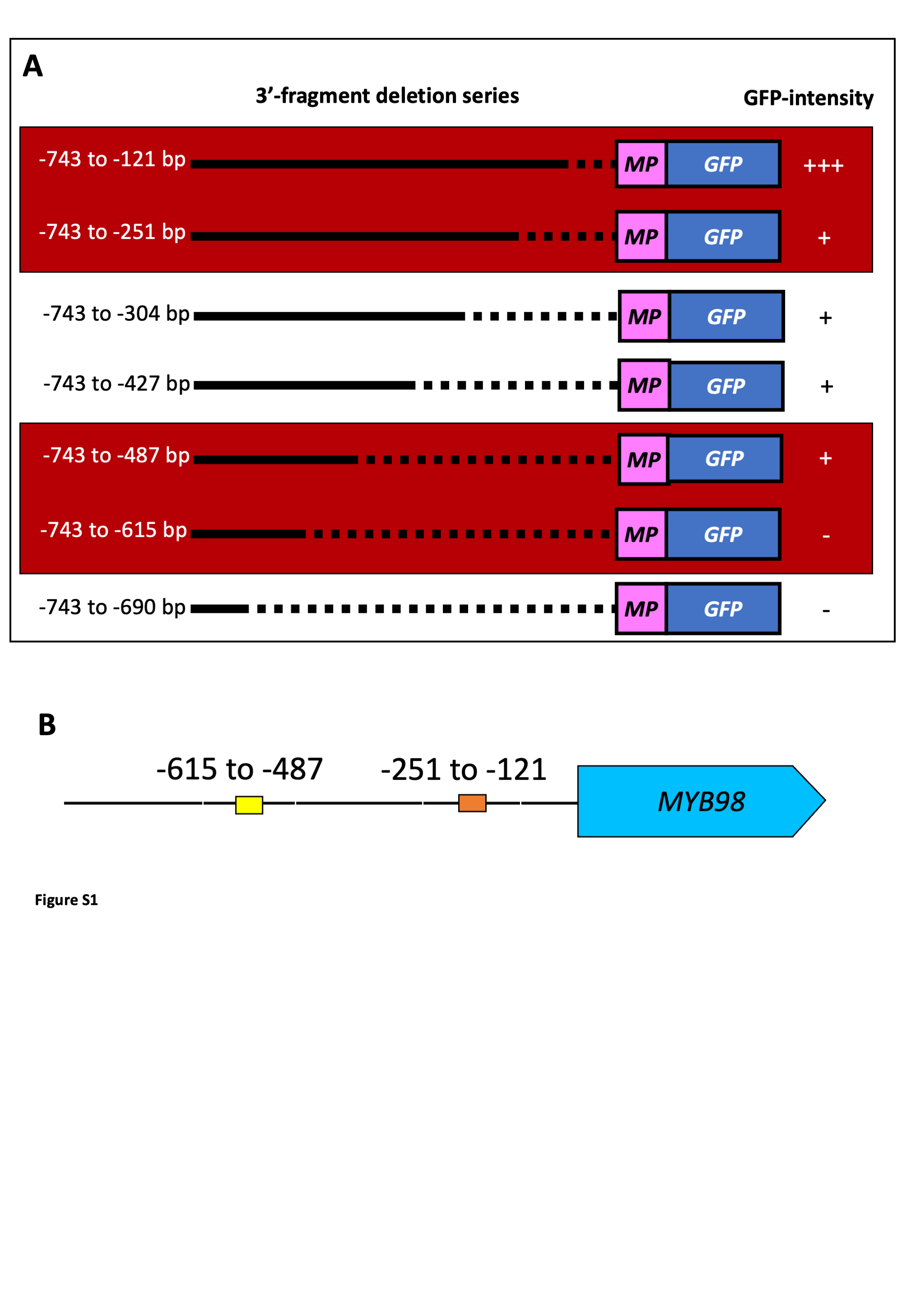

Supplement: Supplementary Figure 7 — Structure and conserved motifs assessment in ANL2 and homologs. (A) Schematic representation showing that ANL2 and HDG1 harbor two EAR motifs in addition to the HD- and START-domains, while HDG7 and ETD1 lack EAR motif. (B) Alignment of ANL2 and homologs with respective conserved motifs are annotated. Green oval circles mark the residues involved in DNA binding and red oval circles mark the residues involved in lipid binding. [file Image_7.tiff]
